# Development and validation of a LC–MS/MS method for quantification of hetrombopag for pharmacokinetics study

**DOI:** 10.1186/s40064-015-1446-0

**Published:** 2015-10-29

**Authors:** Tao Chen, Zhonghua Chen, Suxing Zhang, Kezhi Zhang, Laiyou Wang

**Affiliations:** Guangdong Metabolic Diseases Research Center of Integrated Chinese and Western Medicine, Guangdong TCM Key Laboratory against Metabolic Diseases, Institute of Chinese Medical Sciences, Guangdong Pharmaceutical University, Guangzhou Higher Education Mega Centre, Guangzhou, 510006 People’s Republic of China; Department of Pharmacology, West China School of Pharmacy, Sichuan University, Chengdu, 610041 People’s Republic of China; CarysBio Holdings Limited, Foshan, 528000 People’s Republic of China

**Keywords:** Hetrombopag, Pharmacokinetics, Idiopathic thrombocytopenic purpura, LC–MS/MS

## Abstract

Hetrombopag as the derivative of ethylidene hydrazine carboxamide was recently developed into a novel patented non-peptide thrombopoietin mimetic and thrombopoietin receptor agonist to treat idiopathic thrombocytopenic purpura. To study the pharmacokinetics of hetrombopag, a highly sensitive, rapid and reliable liquid chromatography-tandem mass spectrometry (LC–MS/MS) method was developed and validated for determination of hetrombopag in rat plasma. After protein precipitation extraction, the chromatography separation of analyte and internal standard named eltrombopag as an marketed analog of hetrombopag was performed on an Synergi Polar-RP column at the flow rate of 0.5 mL/min, and the determination was conducted on an API4000 triple quadrupole mass spectrometry in the multiple reaction monitoring mode using the respective [M+H]^+^ ions *m/z* 459.2 → 200.9 for hetrombopag and *m/z* 443.2 → 229.0 for IS. The lower limit of quantification was established to be 1 ng/mL, and the linear scope of standard curve was 1–1000 ng/mL. Both the precision (RSD%) and accuracy (RE%) were within the acceptable criterion of below 15 %. The validated method was successfully applied to quantify hetrombopag in the rat plasma and investigate the pharmacokinetics.

## Background

Idiopathic thrombocytopenic purpura (ITP) involved in anti-platelet antibody mediated platelet destruction and reduced platelet production is a common autoimmune disease in hematology department (Heitink-Pollé et al. 2014; Lo and Deane 2014). ITP usually indicating the persistently low platelet counts less than 20,000 mm^3^ are associated with an increased risk of serious bleeding such as intracranial hemorrhage (Aledort et al. 2004). One of the ITP etiology is that there is no enough thrombopoietin to stimulate platelet production (Kuter 2013). So improving the thrombopoietin concentration level will be great benefit of treating ITP (Imbach and Crowther 2011). Eltrombapag (SB-497115) as thrombopoietin stimulant agent has been approved by FDA and demonstrated that it can significantly increase the platelet counts in relapsed and refractory ITP patients (Bussel et al. 2007; Cheng et al. 2011).

Hetrombopag(SHR8735,(Z)-5-(2-hydroxy-3-(2-(3-methyl-5-oxo-1-(5,6,7,8-tetrahydronaphthalen-2-yl)-1*H*-pyrazol-4(5H)-ylidene)hydrazinyl)phenyl)furan-2-carboxylic acid), the analog of eltrombopag, was developed by Jiangsu Hengrui pharmaceutical company limited as a novel patented thrombopoietin receptor agonist to treat idiopathic thrombocytopenia (Tang et al. 2009). However, some methods have been published for quantification of eltrombopag (Maddela et al. 2014; Deng et al. 2011), there is no reported analytical methods for quantification of hetrombopag. Due to its different structural characteristic and corresponding physicochemical properties of the hetrombopag, it remains to be a challenge to develop a sensitive and selective method for determination of the plasma concentration of this innovative chemical entity. Compared with conventional HPLC methods, the LC–MS/MS method is more sensitive, rapid and applicable (Li et al. 2011). Thus, it is necessary to establish a suitable LC–MS/MS method to accurately quantify the plasma concentration of hetrombopag. In this study, it is the first time to develop and validate a sensitive, specific and reproducible LC–MS/MS method for quantification of hetrombopag in rat plasma. The protein precipitation was used to prepare samples and the eltrombopag was adopted as the internal standard (IS). This analytical method was successfully applied to investigating pharmacokinetics profile of hetrombopag in rat plasma.

## Experimental

### Chemical and materials

Hetrombopag (98.75 %purity), eltrombopag (internal standard (IS) for hetrombopag, 99.29 % purity) were supplied by Jiangsu Hengrui pharmaceutical Co. Ltd (Jangsu, China) Methanol, acetonitrile, dimethyl sulfoxide (DMSO), formic acid of HPLC grade were purchased from Merck (Darmstadt, Germany). High-purity water (18.3 MΩ) was purified by a Millipore Milli-Q Gradient Water Purification System (Millipore, Bedford, USA).

### Instrumentation and conditions

#### Liquid chromatography

Chromatographic analysis was performed using a Shimadzu LC-20AD HPLC system (Shimadzu, Kyoto, Japan) and equipped with the CTC HTS-XT PAL Leap autosampler (Carrboro, NC, USA). The separation column was Synergi Polar-RP column (50 × 2.0 mm, 4 μm, Phenomenex, Torrance, CA). Mobile phase A was water with 0.1 % v/v formic acid, 31 % v/v acetonitrile, 31 % v/v methanol and mobile phase B was methanol with 0.5 % v/v formic acid at a flow rate of 0.5 ml/min according to the following linear gradient: 0–2.50 min, 55 % B; 2.51–3.50 min, 95 % B; 3.51–4.00 min, 55 % B. The injection volume was 20 μL.

#### Mass spectrometry

Samples were analyzed with an API4000 mass spectrometer (AB Sciex, Toronto, Canada) equipped with an ESI source operating in the positive ion (ESI+) mode. The software used for controlling this equipment and analyzing the data was Analyst Version 1.6.1 (AB Sciex, Toronto, Canada). Samples were detected in the multiple reaction monitor (MRM) mode. The mass transitions of the compounds were: hetrombopag, *m/z* 459.2 → 200.9 and eltrombopag (IS), *m/z* 443.2 → 229.0.

The primary parameters of the mass spectrometer were described as follows: ion spray voltage, 4.5 kV; temperature, 500 °C; curtain gas, 30 psi; gas 1 (nebulizer gas) 50 psi; gas 2 (heater gas) 60 psi; collision activate dissociation (CAD) gas 10 psi; collision cell exit potential (CXP), 14 V; declustering potentials (DP): 74 V for hetrombopag, 81 V for IS; collision energies (CE): 41 eV for hetrombopag, 28 eV for IS; dwell time, 150 ms. The gases used were nitrogen.

### Preparation of standard solutions and quality control (QC) samples

The appropriate amount of hetrombopag was accurately and independently weighted in two replicates and dissolved with a small amount of dimethyl sulfoxide (DMSO) firstly, and then dissolved in methanol to produce the standard stock solutions at the concentration of 1 mg/mL, respectively. The final DMSO concentration was around 0.1 %. One of standard stock solutions was prepared for standard solutions, and the other was prepared for QC samples. A serial of hetrombopag standard working solutions were produced by diluting standard solutions with methanol–water (v/v, 50:50) to obtain the desired concentration. The calibration standards were prepared by spiking 50 μL standard working solutions to 50 μL drug-free rat plasma at the concentrations of 1, 3, 10, 30, 100, 250, 500, 750, 1000 ng/mL. The QC samples were produced by diluting standard solutions with drug-free rat plasma at the concentrations of 2, 150, 600, 800 ng/mL. The similar stock solution of IS was diluted at the concentration of 700 ng/mL working solution. These solutions were all stored at 4 °C and then brought to room temperature before use.

### Sample preparation

All the frozen rat plasma samples were thawed at room temperature and vortexed thoroughly. A 50 μL aliquot of IS and 50 μL methanol–water (v/v, 50:50) were added to 50 μL plasma sample with a 96-well plate. Then 200 μL methanol were added to the sample for precipitation. The sample was vortex-mixed for 15 min, and centrifuged 3220*g* for 20 min under 15 °C. The supernatant (20 μL) was then injected into the LC–MS/MS system for analysis.

### Method validation

Method validation was performed based on the US Food and Drug Administration (FDA) guideline for industry bioanalytical method validation (FDA 2001) and European Medicines Agency (EMA) guideline (EMA 2009). The validation was assessed included specificity, linearity, inter- and intra-precision and accuracy, extraction recovery, matrix effect and stability.

#### Specificity

The selectivity was evaluated by analyzing 6 drug-free plasma samples and 12 spiked plasma samples at the lower limit of quantification (LLOQ) level from six different rat plasma sources. The MRM chromatograms of the blank plasma samples were compared with the corresponding spiked plasma samples with analyte and IS. The peak areas of the endogenous compounds co-eluted with the analytes should be ≤20 % of the peak area of the LLOQ standard.

#### Linearity and LLOQ

The linearity was estimated by assaying calibration curves in rat plasma in duplication on three consecutive days. The calibration curves were fitted by a weighted (1/x^2^) least squares linear regression method and its acceptable criterion was that correlation coefficient (r) was ≥0.995.

The LLOQ defined as the lowest concentration of the calibration curve was determined in six replicates in three validation runs. Compared with the nominal concentration, the accuracy was within ±20 % and the precision was ≤20 %.

#### Accuracy and precision

Accuracy and precision were evaluated by determining the concentrations of QC samples at four levels using six replicates on three validation runs. The relative error (RE%) was used to assessing the accuracy and relative standard deviation (RSD%) was used to assessing the precision. The acceptable criterion of the inter-day and intra-day precision was less than 15 %, and the accuracy was within ±15 %.

#### Dilution reliability

A sample was spiked at 8000 ng/mL for hetrombopag and diluted by blank plasma to 10-fold, 25-fold and 50-fold in six replicates, respectively. The concentrations of diluted samples were all over the calibration curve. Compared with the concentration of samples before dilution, the accuracy should be between 85 and 115 %, and relative error should be less than 15 %.

#### Extraction recovery and matrix effect

Extraction recovery was assessed at three concentration levels by comparing peak areas of analyte and IS in six replicate QC samples with those of post-extracted blank plasma spiked at corresponding concentrations. The post-extracted samples represented 100 % recovery.

Matrix effect (ME) was conducted at low and high concentration levels by comparing peak areas of analyte and IS in triplicate with those at the same concentration solutions. The drug-free rat plasma from six different donors were precipitated and then spiked with analyte and IS, the peak areas of which was defined as A. Those of the water-substituted samples at same concentrations were defined as B. The ratio (A/B × 100 %) was defined as matrix factor (MF) for investigating ME. The RSD% of IS-normalized matrix factors should be less than 15 %.

#### Stability

The stability of hetrombopag in rat plasma was assessed by determining the low QC and high QC samples in triplicate under different conditions. The short-term stability was determined via analyzing the QC samples exposed to the room temperature for 4 h. The samples after protein precipitation were exposed to the autosampler for 24 h in room temperature to estimate the post-preparative stability. Freeze–thaw stability was evaluated after 3 freeze–thaw cycle of the QC samples from −80 °C to ambient temperature on consecutive days, and the long-term stability was assessed at the −80 °C for 45 days. The acceptable criteria of the stability was within ±15 % of the accuracy.

### Pharmaceutical study

The validated analytical method was successfully applied to pharmaceutical study of hetrombopag in rats (9 females, 9 males) purchased form Guangdong Province Experimental Animal Center. The procedure about this animal experiment was approved by the animal ethics committee of Guangdong Pharmaceutical University. Hetrombopag (10, 30, 60 mg/kg) was oral administrated by intragastric administration and blood samples were collected into EDTA.2K tubes from the retrobulbar venous plexus at before dosing and at 0.25, 0.5, 1, 2, 4, 6, 8, 12, 24, 48 h after dosing. The plasma samples were separated through centrifuge immediately and stored at −80 °C until analysis. Software Kinetica 4.4.1 (USA, Thermo Electron Corporation Company) was adopted for the pharmacokintic analysis.

## Results and discussion

### Optimization of mass spectrometric condition

**Fig. 1:**
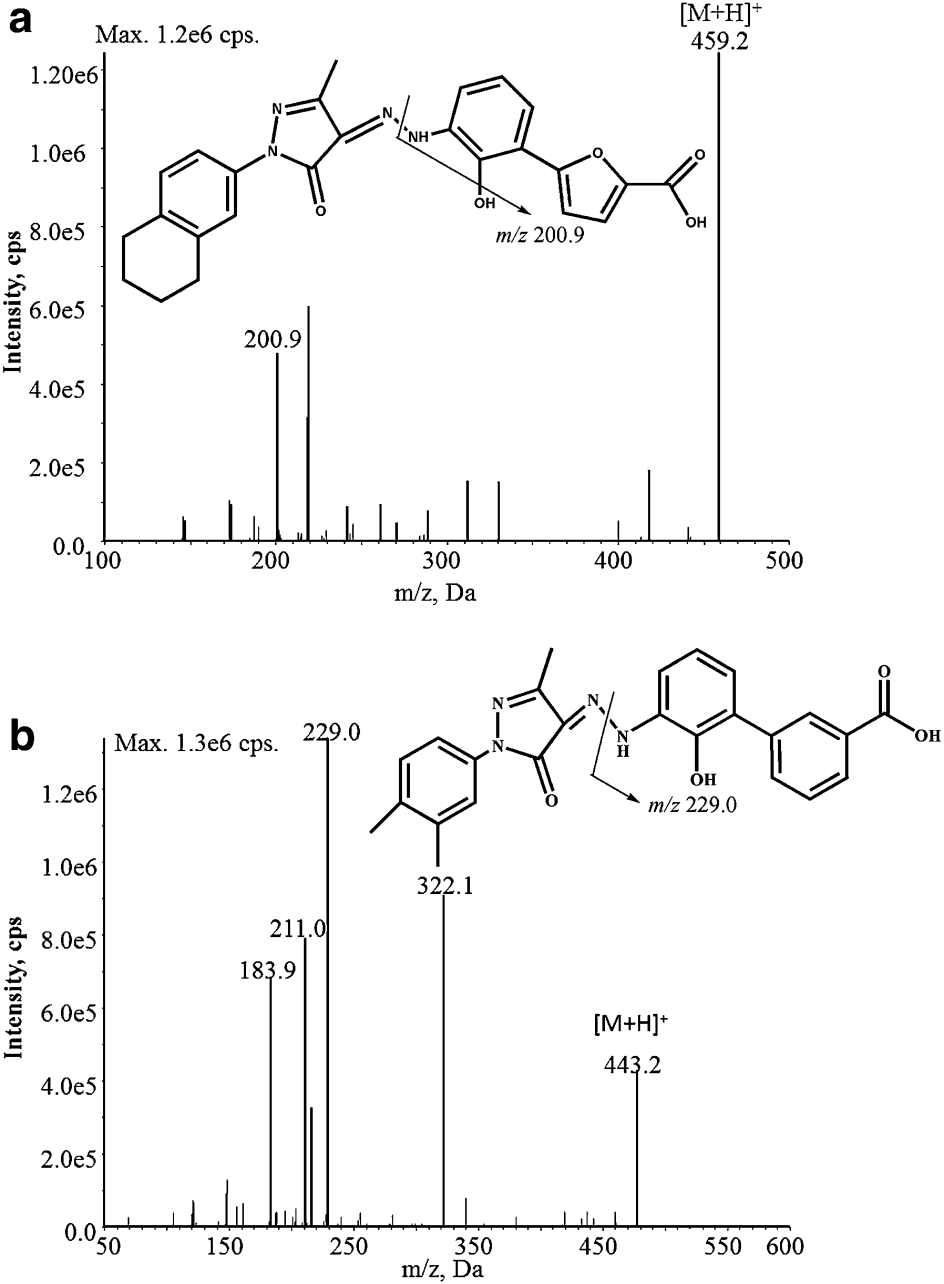
Production mass spectra of [M+H]^+^ ion of **a** hetrombopag, and **b** eltrombopag

The mass spectrometry parameters were optimized by directly infusing the 1 μg/mL standard solution of hetrombopag and eltrombopag into the mass spectrometry, respectively. Taking the signal response into consideration, the positive mode was chosen to quantify hetrombopag. Under the positive ESI conditions, hetrombopag and eltrombopag produced predominantly protonated molecule [M+H]^+^ at *m/z* 459.2 and *m/z* 443.2 respectively in Q1 full scan mass spectra. The corresponding product ion mass spectra were showed in Fig. 1, where [M+H]^+^ of each compound was selected as precursor ion. Hetrobopag produced primarily fragment ions at *m/z* 219.0 and 200.9, and we chosen MRM transition *m/z* 459.2 → 200.9 for quantification analysis due to its more stable and less disturbed presence than the *m/z* 459.2 → 219.0. Similarly, the most abundant and stable fragment ions at *m/z* 443.2 → 229.0 was selected as the transition for IS.

### Chromatography

In order to obtain a suitable chromatography, a lot of commercially available column were assessed including Synergi hydro-RP C18, Gemini C18, Kinetex C18 and Synergi Polar-RP. In respect of the peak shape, retention time, sensitivity, carry-over and baseline noise for analyte and IS, the Synergi Polar-RP column (50 × 2.0 mm, 4 μm) column was found to be optimal, which exhibited good peak shape and retention time. As the hetrombopag was hard to be eluted and the carry-over effect was observed, the composition of mobile phases was also investigated. As a consequence, methanol–acetonitrile with formic acid was optimal mobile phase. Using the gradient elution, the analytical run time was 4 min, and the retention time for hetrombopag and IS were 1.5 and 1.3 min, respectively.

### Method validation

#### Specificity

**Fig. 2:**
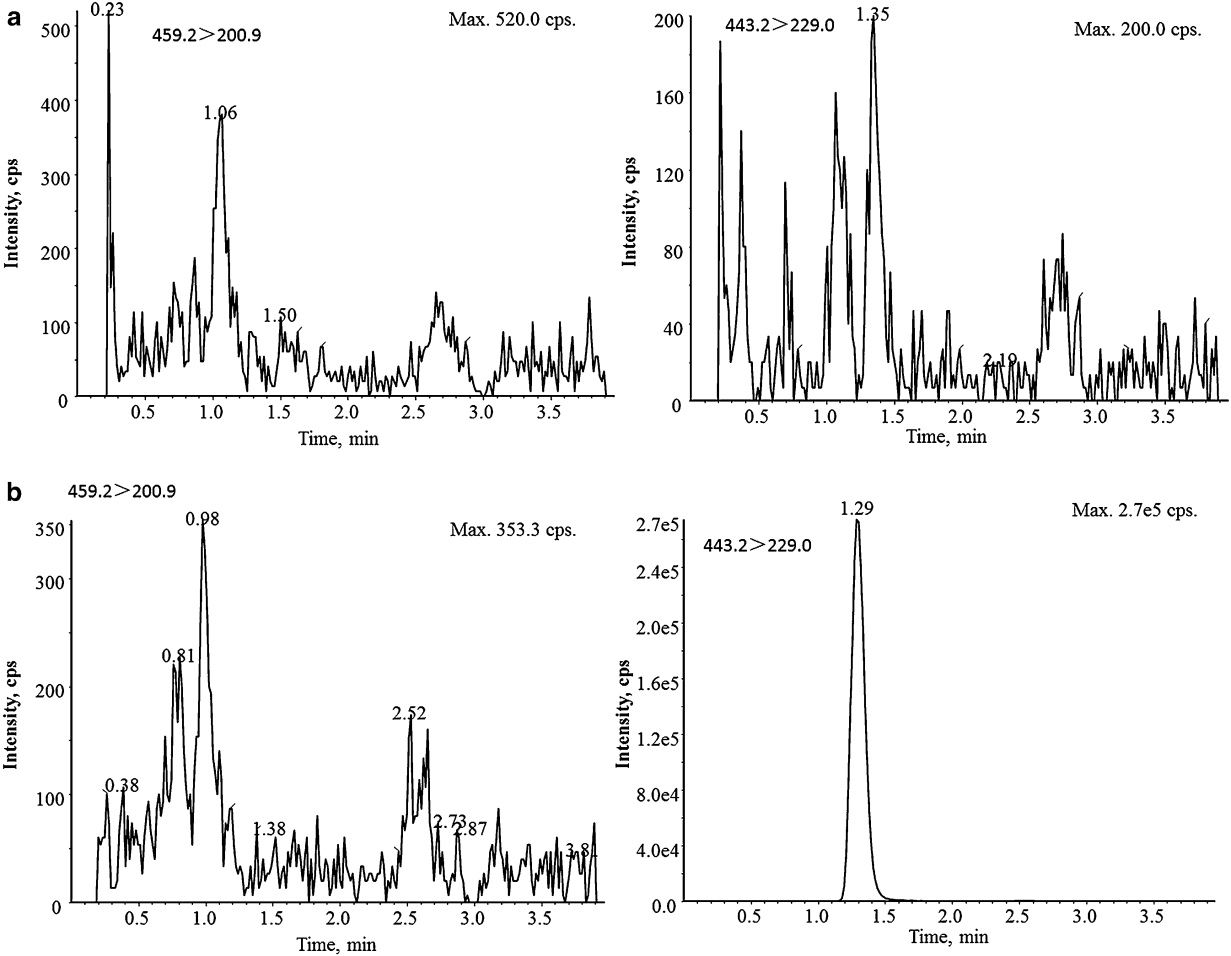
Typical chromatograms of hetrombopag and its corresponding internal standard in rat plasma. **a** double blank plasma (drug and IS free); **b** blank plasma (drug free and spiked with 700 ng/mL IS)

**Fig. 3:**
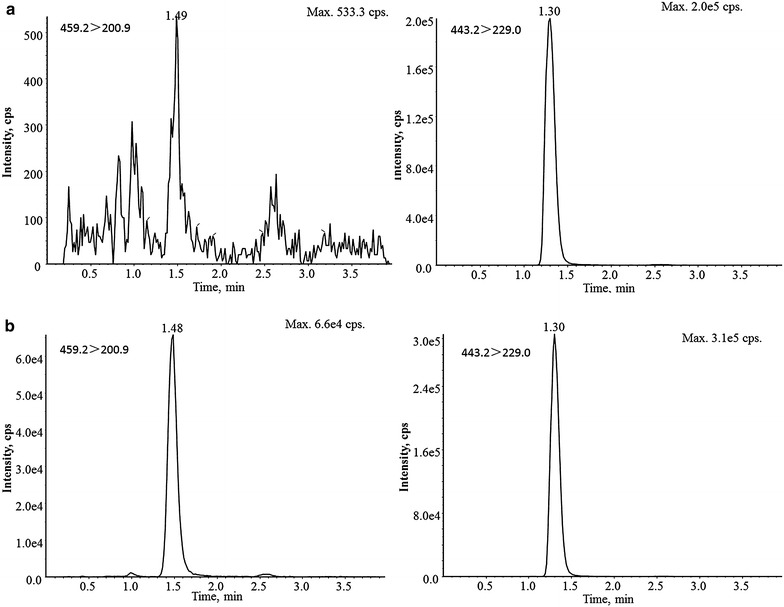
Typical chromatograms of hetrombopag and its corresponding internal standard in rat plasma. **a** LLOQ of hetrombopag in rat plasma (spiked with 1 ng/mL hetrombopag and 700 ng/mL combined IS; **b** plasma sample 0.25 h after intragastric administration of 60 mg/kg hetrombopag ethanolamine tablets

The results of typical chromatograph shown in Figs. 2, 3 indicated that the response of endogenous compounds co-eluted with analyte was within 20 % of the LLOQ standard, which demonstrated that the endogenous compounds had no effect on the quantification of hetrombopag under current conditions.

#### Linearity and LLOQ

The calibration curve of concentration of hetrombopag over the ranges of 1–1000 ng/mL was established by weighted (1/x^2^) linear regression analysis, and the coefficient correlation (R) was ≥0.995. The typical equation of hetrombopag calibration curve was y = 0.00204x + 0.0000939 (r = 0.9987), where y represents the ratio of the peak area of the analyte to that of IS, and x represents the plasma concentration.

The LLOQ of hetrombopag in rat plasma was 1 ng/mL, the sensitivity was enough to determine the plasma concentration and investigate the pharmacokinetics of hetrombopag in rat. The mean inter-and intra-assay precision (RSD%) at LLOQ concentration level were 11.3 and 5.6 %, respectively, with relative error 5.4 %, which was described in Table 1.

**Table 1:** Precision and accuracy data for hetrombopag in rat plasma (3 days with 6 replicates per day)

Sample name	Concentration (ng/mL)		RSD (%)		RE (%)
	Nominal	Calculated	Intra-run	Inter-run	
LLOQ	1.0	1.1	11.3	5.6	5.4
QC4	2.0	2.1	9.7	3.9	2.5
QC3	150.0	151.5	2.6	4.1	1.0
QC2	600.0	605.2	2.7	2.5	0.9
QC1	800.0	770.0	2.7	8.7	−3.8

#### Precision and accuracy

The inter- and intra-assay precision and accuracy values of QC samples were depicted in Table 1. The values of inter- and intra-assay mean precision were less than 9.7 and 8.7 %, respectively, and the mean values of accuracy were between −3.8 and 2.5 %, which revealed that the current method was reliable, accurate and reproducible.

#### Dilution reliability

The results of dilution factor (DF) showed in Table 2, and the values of precision and accuracy were all in the acceptable ranges, which were ≤2.1 and ≤11.7 %, respectively.

**Table 2:** Dilution factor (DF) for hetrombopag (n = 6)

Sample name	Concentration(ng/mL)		RSD (%)	RE (%)
	Nominal	Calculated		
DF 50-fold	160.0	178.7	2.1	11.7
DF 25-fold	320.0	326.8	1.4	2.1
DF 10-fold	800.0	811.5	1.5	1.4

#### Matrix effect and extraction recovery

The matrix factors from six different lots of blank plasma ranged from 67.6 to 91.8 % for analyte and IS. The RSD of the inter-subject variability of the IS-normalized MF was less than 13.7 %. The results indicated that the ion-enhancement or suppression from rat plasma matrix were negligible under current method.

The mean extraction recoveries of hetrombopag from three different QC levels were 88.1, 85.0, 91.0 %, respectively. The mean extraction recovery of IS was 75.2 %. The results demonstrated that the extraction process is efficient.

#### Stability

The detailed results of stability showed in Table 3. The results showed that the analyte of samples was stable after exposed to the room temperature for 4 h, 3 freeze–thaw cycle −80 °C to ambient temperature on consecutive days and stored at −80 °C for 45 days. The samples after extraction stored at the autosampler for 24 h in room temperature remained to be stable.

**Table 3:** Stability of hetrombopag in rat plasma under various storage conditions (n = 3)

Storage conditions	Concentration (ng/mL)		RSD (%)	RE (%)
	Nominal	Calculated		
Long-term (−80 ℃ 45 days)	2	2.02	4.5	1.2
	800	700	2.6	−12.5
Short-term (room temperature 4 h)	2	2.12	5.2	5.8
	800	713	2.9	−10.9
Three freeze/thaw cycles (−80 ℃)	2	2.17	6	8.5
	800	710	5.3	−11.3
Post-preparative (room temperature 24 h)	2	2.18	5.8	9.2
	800	726	6	−9.2

### Pharmacokinetic study

**Fig. 4:**
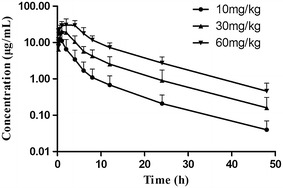
Mean drug plasma concentration–time curve of hetrombopag from 18 rats (three levels doses) after intragastric administration

The assay depicted in this study was successfully applied to investigate the pharmacokinetic profiles of hetrombopag in rats following intragastrical administration of 10, 30, 60 mg/kg hetrombopag ethanolamine tablets. Figure 4 demonstrated the plasma concentration–time curves, and the main pharmacokinetic parameters for hetrombopag were presented in Table 4. The t_1/2_ values for hetrombopag was approximately 8–9 h, and the C_max_ (µg/mL) were 13.1 ± 6.0, 21.6 ± 12.3, 34.6 ± 11.9 after intragastrical administration of 10, 30, 60 mg/kg hetrombopag, respectively. To our knowledge, this is the first time to report the pharmacokinetics parameter of hetrombopag analogs in rats.

**Table 4:** The main pharmacokinetic parameters for hetrobopag after intragastric administration of 10, 30 and 60 mg/kg to rat, respectively (mean ± SD, n = 6)

Parameters	10 mg/kg	30 mg/kg	60 mg/kg
C_max_ (µg/mL)	13.1 ± 6	21.6 ± 12.3	34.6 ± 11.9
T_max_ (h)	0.7 ± 0.26	1.67 ± 0.52	2.58 ± 2.06
AUC_0–48 h_ (µg h/mL)	46.2 ± 31	127.7 ± 70.8	306.6 ± 94.6
AUC_0–∞_ (µg h/mL)	46.7 ± 31.3	129.8 ± 72.8	312.8 ± 98.8
MRT (h)	6.5 ± 0.7	8.4 ± 1.7	105 ± 1.3
t_1/2_ (h)	8.5 ± 1.1	8.4 ± 1.1	8.8 ± 1.1

## Conclusion

A highly selective LC–MS/MS method was successfully developed and validated for determination of hetrombopag in rat plasma for the first time. The analytical time of this method was 4 min, which was more rapid than conventional HPLC method, and a highly sensitive was observed in this method (LLOQ for hetrombopag was 1 ng/mL). A simple and quick protein precipitation was used to extract the samples prior to LC–MS/MS analysis. The method exhibited good precision, accuracy and wide linear concentration range. Hence, the current method could be suitable for providing sufficient support for further preclinical study in pharmacokinetics investigation.
